# Management of pseudoaneurysm and cervical hematoma following carotid sheath placement in a patient with middle cerebral artery thrombosis: a case report

**DOI:** 10.1186/s13256-025-05531-5

**Published:** 2025-10-01

**Authors:** Meghdad Ghasemi Gorji, Rojan Abdollahzadeh Mirali, Yasamin Bigdeli

**Affiliations:** 1 Department of Vascular Surgery, Shiraz University of Medical Science, Shiraz, Iran; 2 Student Research Committee, Shiraz University of Medical Sciences, Shiraz, Iran; 3 Student Research Committee, Faculty of Medicine, Qom University of Medical Sciences, Qom, Iran

**Keywords:** Mechanical thrombectomy, Middle cerebral artery, Carotid sheath placement, Cervical hematoma, Pseudoaneurysm

## Abstract

**Background:**

Middle cerebral artery occlusions are present in up to one third of patients with acute ischemic strokes who are undergoing endovascular mechanical thrombectomy, which, especially with intravenous thrombolysis, effectively treats proximal middle cerebral artery stroke when performed promptly. However, carotid sheath placement carries risks such as pseudoaneurysm and hematoma.

**Case presentation:**

This report describes a 73-year-old male of Iranian ethnicity with right middle cerebral artery occlusion who developed a pseudoaneurysm and cervical hematoma after carotid sheath placement during mechanical thrombectomy. Despite these complications, the patient underwent successful surgical repair and recovered fully.

**Conclusion:**

This case emphasizes the importance of timely middle cerebral artery thrombectomy and highlights the potential complications of carotid sheath placement. It underscores the need for timely management to minimize risks and achieve optimal outcomes.

## Introduction

Middle cerebral artery (MCA) occlusions are present in up to one third of patients with acute ischemic strokes undergoing endovascular mechanical thrombectomy (MT) [1]. Strokes involving the MCA often result in severe neurological deficits owing to the MCA’s extensive vascular distribution [2]. Prompt and effective surgical intervention is vital when dealing with MCA blockage, as delays can lead to irreversible brain damage [3].

Advances in surgical techniques, such as MT, have revolutionized the management of acute ischemic stroke in the proximal MCA. When combined with intravenous thrombolysis, thrombectomy has been shown to be highly effective if performed within 6 hours of symptom onset. Surgical precision during MT is crucial to minimize risks and optimize outcomes [4].

However, invasive vascular procedures, such as carotid endarterectomy or arterial sheath placement, are not without complications. Surgical manipulation of the carotid artery can result in adverse events, including pseudoaneurysms, hematomas, or arterial rupture [5]. Direct arterial access and sheath placement during MCA thrombosis treatment, while essential, can also lead to significant complications at the access site [6].

This case report discusses a patient who developed a pseudoaneurysm and cervical hematoma following carotid sheath placement during the surgical management of MCA thrombosis and highlights strategies for managing these complications effectively.

## Case presentation

A 73-year-old male of Iranian ethnicity with a history of hypertension and diabetes mellitus presented to the emergency department. He experienced sudden left-sided weakness (hemiplegia), difficulty speaking (aphasia), visual disturbances, and confusion. A non-contrast head computed tomography (CT) scan revealed a hyperdense MCA sign, and subsequent CT angiography confirmed a right MCA occlusion. The patient was immediately transferred to the interventional radiology suite for emergency MT. Initially, the procedure was attempted via a retrograde femoral approach but was unsuccessful owing to the tortuosity of the femoral artery. A carotid sheath was then placed in the right common carotid artery to facilitate the procedure. Successful reperfusion of the MCA was achieved, and a significant improvement in neurological function was observed.

**Fig. 1:**
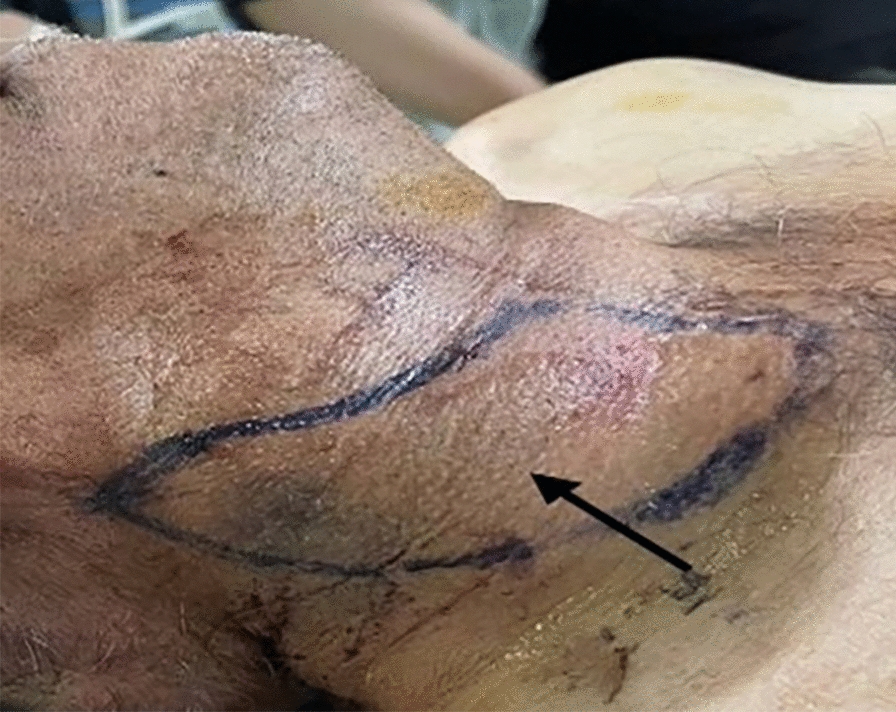
Arrow indicates hematoma on the right side of the neck

**Fig. 2:**
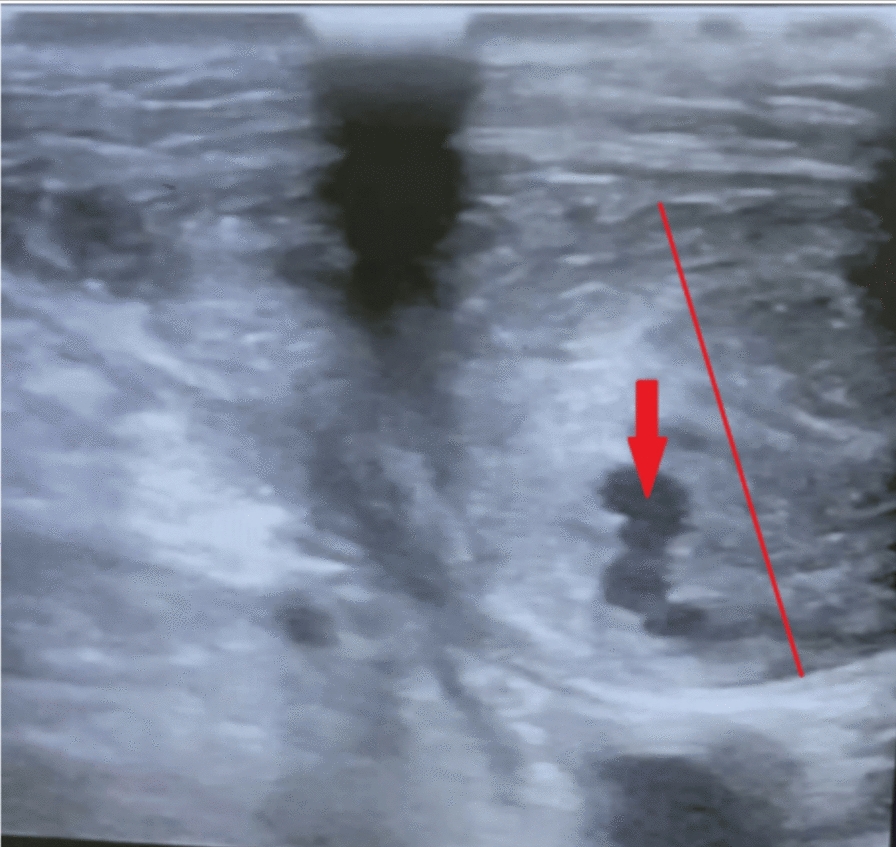
Arrow indicates pseudoaneurysm on the right side of the neck. The red line highlights the hematoma surrounding the pseudoaneurysm

However, immediately after the procedure and removal of the carotid sheath, the patient developed bleeding and a large hematoma in the carotid region, accompanied by a feeling of fullness (Fig. 1). Physical examination revealed a tense, pulsating mass in the right neck area. Femoral angiography was performed, but despite the hematoma, no vascular extravasation (leakage) was detected. An emergency neck Doppler ultrasound showed a 10 mm pseudoaneurysm at the carotid sheath insertion site and a 50 mm × 20 mm neck hematoma adjacent to it (Fig. 2).

Owing to the risk of rupture and potential airway obstruction, a multidisciplinary team, including vascular surgeons, interventional radiologists, and otolaryngologists, was consulted. The patient was immediately taken to the operating room for repair and hematoma evacuation. Under general anesthesia, a longitudinal incision was made along the anterior border of the sternocleidomastoid muscle. The hematoma was evacuated, and the pseudoaneurysm was identified. Direct surgical repair of the pseudoaneurysm was performed using a patch angioplasty technique.

The patient had an uneventful recovery. A repeat ultrasound on the second day postoperation showed no residual pseudoaneurysm or significant hematoma. The patient was discharged on the fourth day postoperation with close outpatient follow-up instructions.

## Discussion

Middle cerebral artery (MCA) occlusions causing acute ischemic stroke present significant clinical challenges. The optimal treatment approach remains unclear. Mechanical thrombectomy (MT) poses unique challenges in these cases, and successful outcomes with MT are rarely documented [7].

Treating complex MCA aneurysms and pseudoaneurysms is challenging but can lead to favorable outcomes with proper surgical strategies. A protective bypass, established distal to the site, ensures adequate blood flow during repair and revascularization while minimizing risks such as hematoma formation [8]. Angioplasty, however, is less invasive and ideal for anatomically challenging cases, but it but may not provide definitive repair for certain conditions [1].

A study reported that in two patients (8%), in-stent thrombosis occurred intraoperatively. However, 2 weeks after discharge, one patient developed a hematoma, which was treated with surgical drainage. Digital subtraction angiography (DSA) was performed in 16 patients (64%), identifying 21 aneurysms (67%) [9].

Using patch angioplasty is a favorable choice for repairing pseudoaneurysms in anatomically challenging locations, including the MCA [10]. There have been reported cases where the adventitia or surrounding soft tissue is affected, and in such cases, patch angioplasty offers an effective repair. This approach is especially beneficial when other treatment options may pose greater risks, as observed in complex vascular injuries [1, 10, 11].

## Conclusion

Managing MCA aneurysms and pseudoaneurysms requires specialized surgical approaches, with protective bypass ensuring blood flow and minimizing complications such as hematoma. Patch angioplasty is a favorable option for repairing pseudoaneurysms in anatomically difficult areas such as the MCA, particularly when other treatments carry higher risks. Further research is needed to refine these strategies.

## Data Availability

You may contact the corresponding author for further case information if interested. You may also visit www.vascularsurgery.ir for more detailed videos and photos of this case.

## Abbreviations

MCA: Middle cerebral artery
MT: Mechanical thrombectomy
